# Supplementary figures and images for: Protein Kinase C promotes peroxisome biogenesis and peroxisome–endoplasmic reticulum interaction
Source: J Cell Biol. 2025 Jul 21;224(9):e202505040. doi: 10.1083/jcb.202505040 (PMC12278821; doi:10.1083/jcb.202505040)

Source Data Figure 1

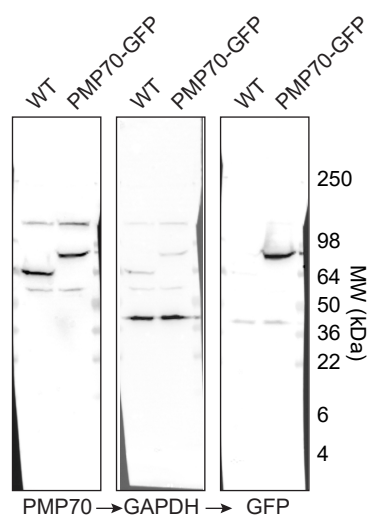

Supplement: SourceData F1 — is the source file for Fig. 1. [file jcb_202505040_sourcedataf1.pdf]

Source Data Figure 2

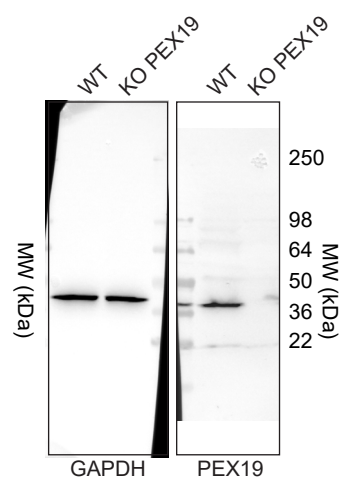

Supplement: SourceData F2 — is the source file for Fig. 2. [file jcb_202505040_sourcedataf2.pdf]

Source Data Figure 3

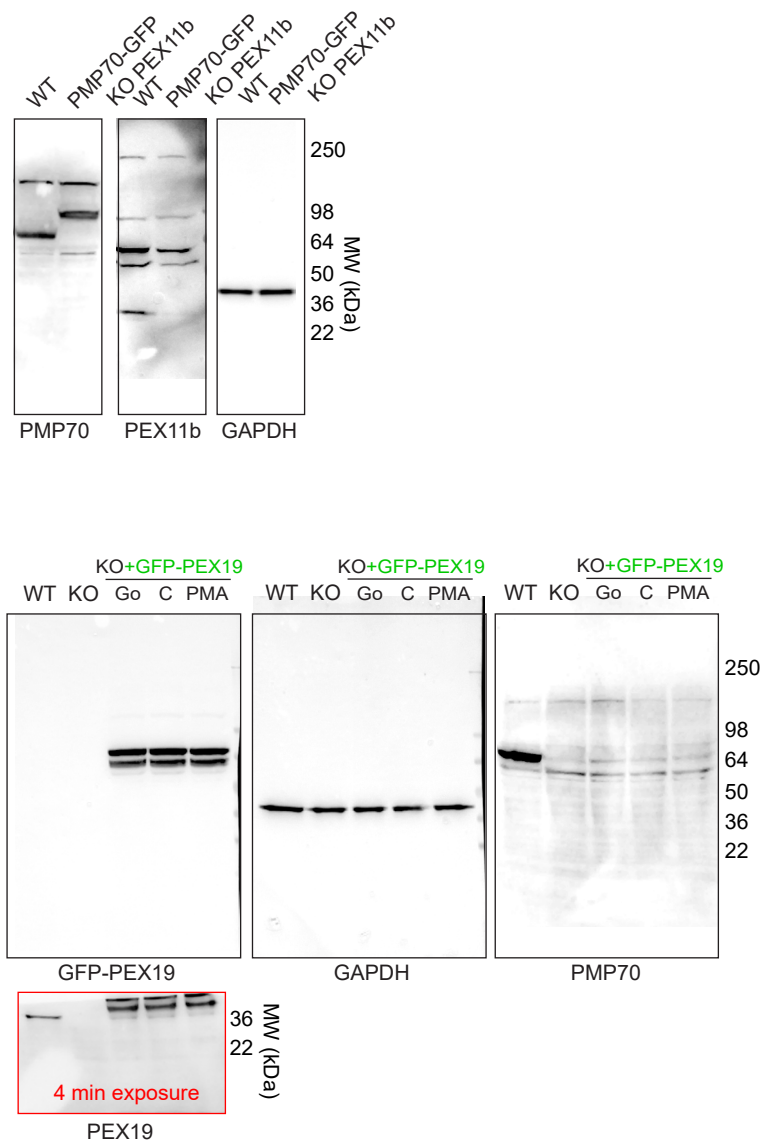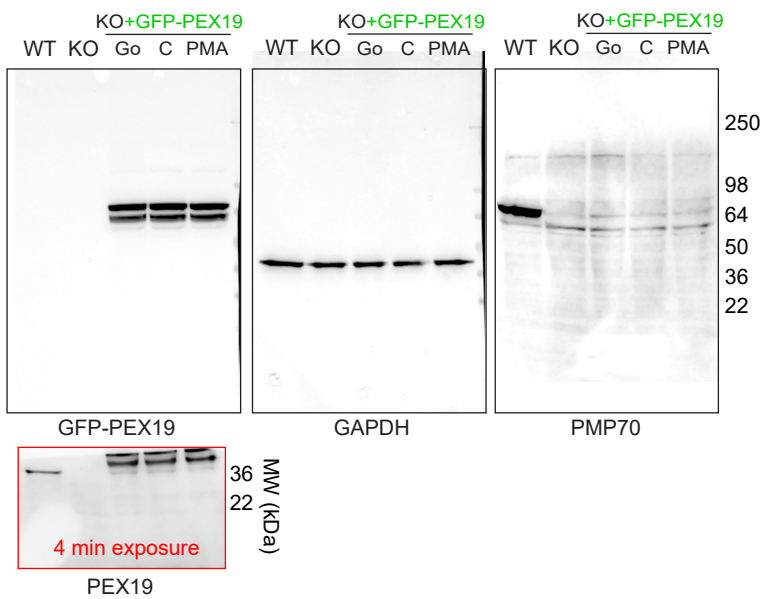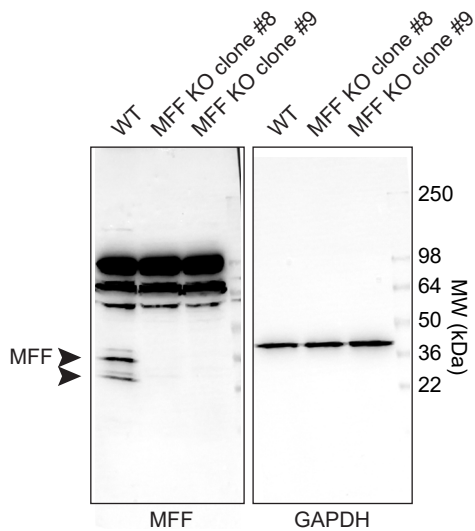

Supplement: SourceData F3 — is the source file for Fig. 3. [file jcb_202505040_sourcedataf3.pdf]

Source Data Figure 4

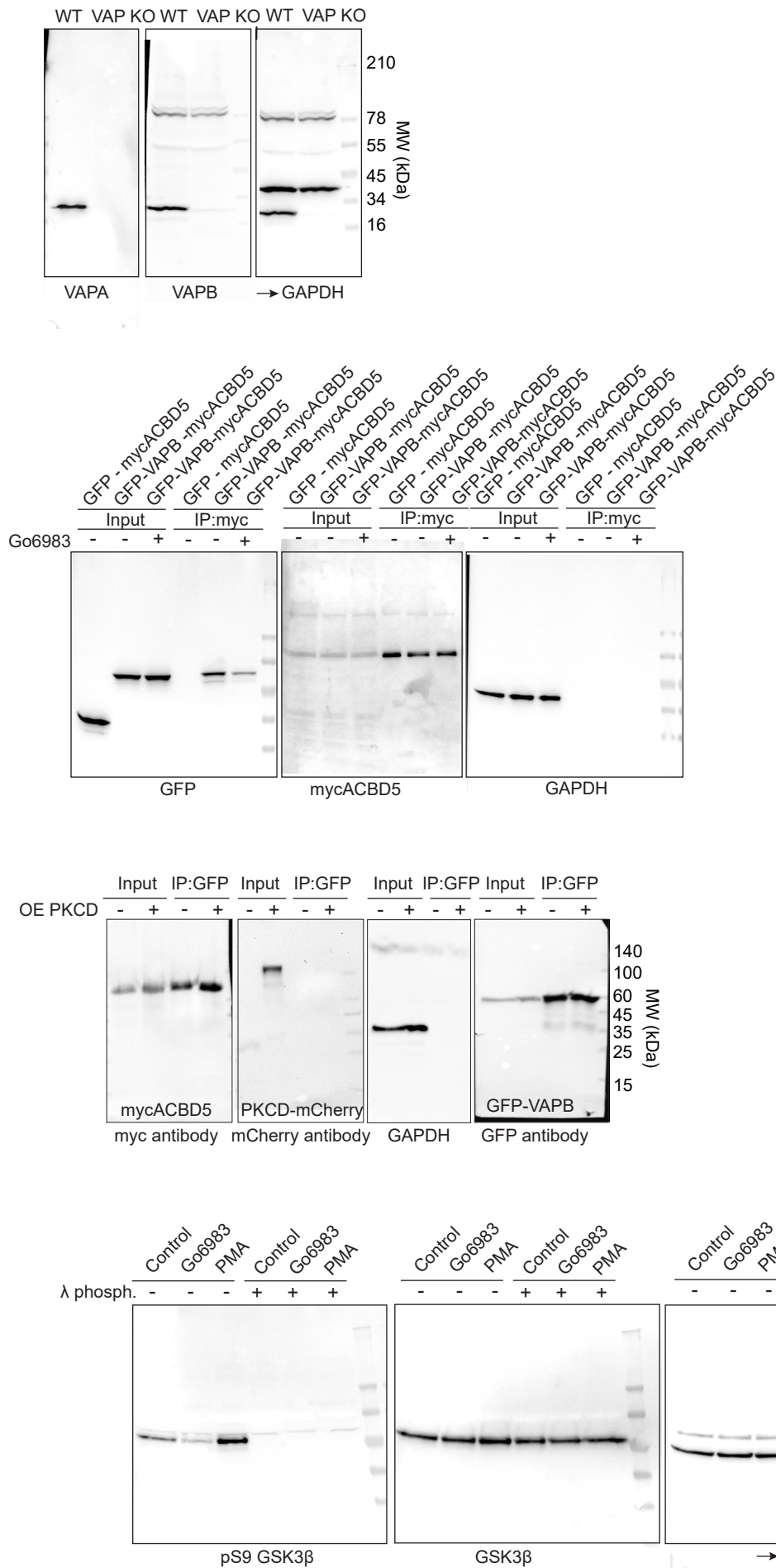

Supplement: SourceData F4 — is the source file for Fig. 4. [file jcb_202505040_sourcedataf4.pdf]

Source Data Figure 5

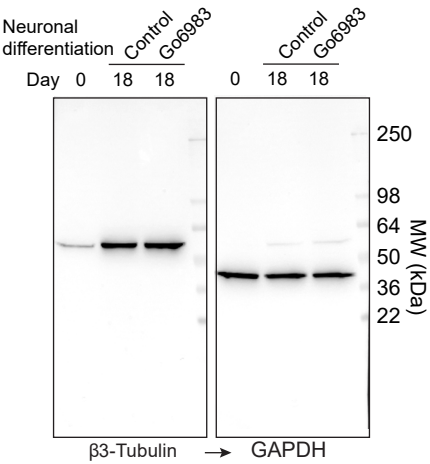

Supplement: SourceData F5 — is the source file for Fig. 5. [file jcb_202505040_sourcedataf5.pdf]

Source Data Figure S1

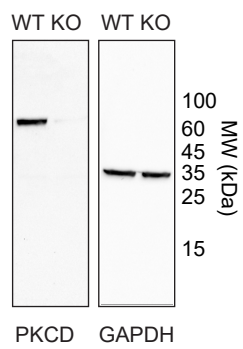

Supplement: SourceData FS1 — is the source file for Fig. S1. [file jcb_202505040_sourcedatafs1.pdf]

Source Data Figure S4

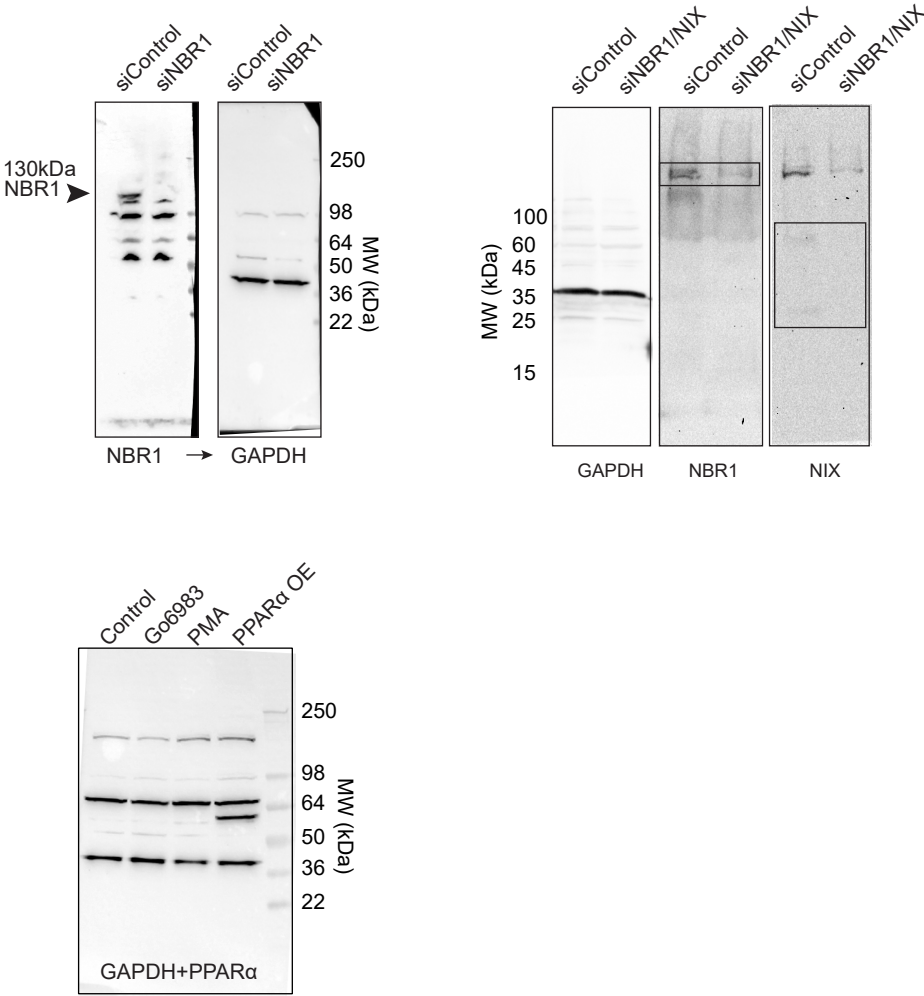

Supplement: SourceData FS4 — is the source file for Fig. S4. [file jcb_202505040_sourcedatafs4.pdf]

Source Data Figure S5

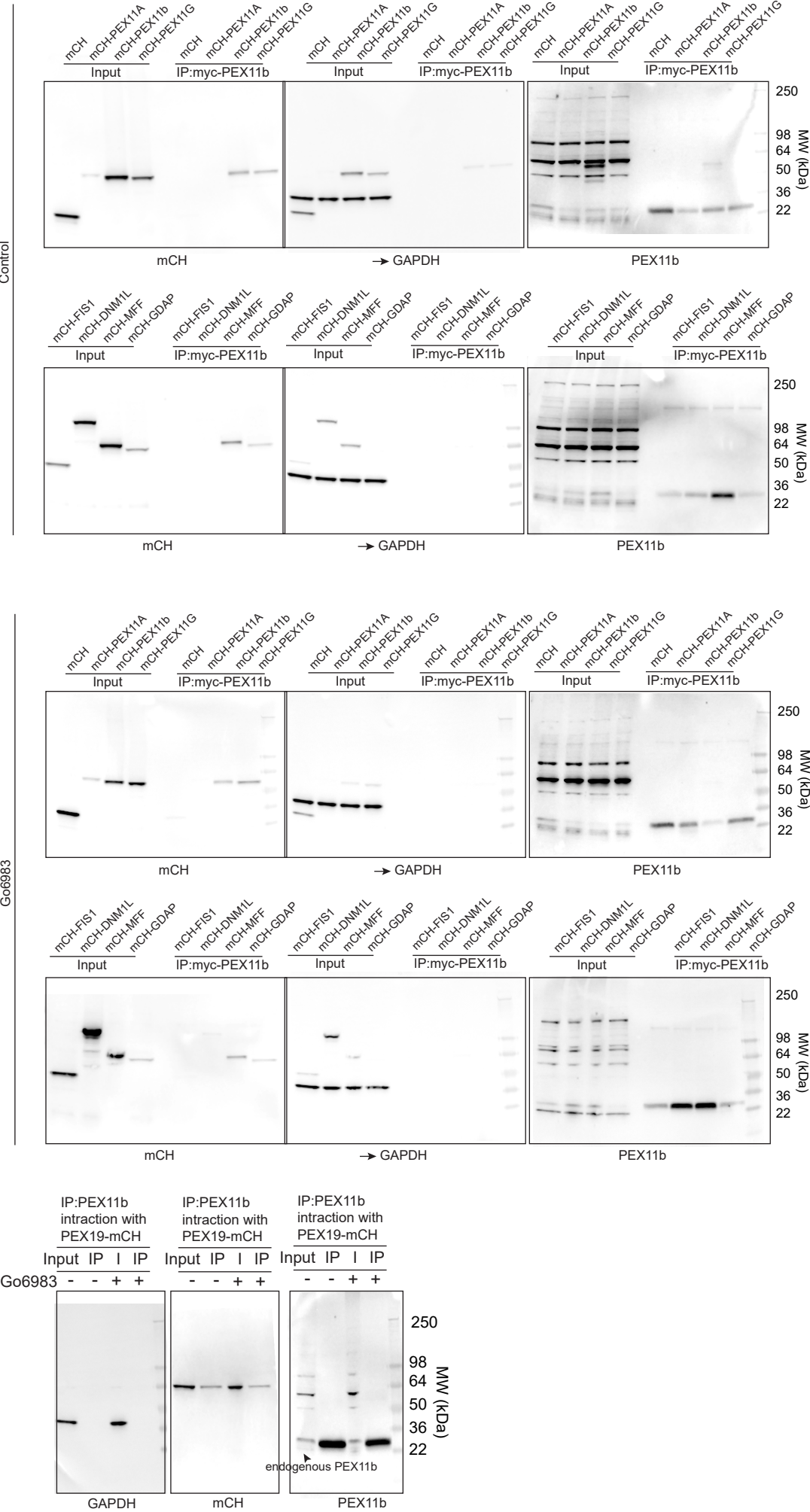

Supplement: SourceData FS5 — is the source file for Fig. S5. [file jcb_202505040_sourcedatafs5.pdf]

Source Data Figure S6

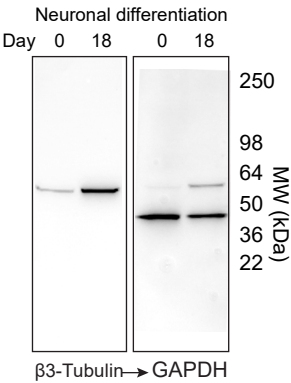

Supplement: SourceData FS6 — is the source file for Fig. S6. [file jcb_202505040_sourcedatafs6.pdf]
